# The hierarchically organized splitting of chromosomal bands for all human chromosomes

**DOI:** 10.1186/1755-8166-2-4

**Published:** 2009-01-26

**Authors:** Nadezda Kosyakova, Anja Weise, Kristin Mrasek, Uwe Claussen, Thomas Liehr, Heike Nelle

**Affiliations:** 1 Universitätsklinikum Jena, Institut für Humangenetik und Anthropologie, Jena, Germany

## Abstract

**Background:**

Chromosome banding is widely used in cytogenetics. However, the biological nature of hierarchically organized splitting of chromosomal bands of human chromosomes is an enigma and has not been, as yet, studied.

**Results:**

Here we present for the first time the hierarchically organized splitting of chromosomal bands in their sub-bands for all human chromosomes. To do this, array-proved multicolor banding (aMCB) probe-sets for all human chromosomes were applied to normal metaphase spreads of three different G-band levels. We confirmed for all chromosomes to be a general principle that only Giemsa-dark bands split into dark and light sub-bands, as we demonstrated previously by chromosome stretching. Thus, the biological band splitting is in > 50% of the sub-bands different than implemented by the ISCN nomenclature suggesting also a splitting of G-light bands. Locus-specific probes exemplary confirmed the results of MCB.

**Conclusion:**

Overall, the present study enables a better understanding of chromosome architecture. The observed difference of biological and ISCN band-splitting may be an explanation why mapping data from human genome project do not always fit the cytogenetic mapping.

## Background

The biological nature of hierarchically organized splitting of bands of human chromosomes remained an enigma since the first banding methods were described in 1970 and 1971. The technique introduced by Lore Zech in Caspersson's laboratory involved quinacrine mustard (Q-banding) and fluorescence microscopy [1], while other used Giemsa (G-banding) [2,3]. Though several methods producing chromosome bands were developed later, G-banding became the one most widely used. A uniform system of human chromosomal nomenclature, which allowed to design not only individual chromosomes but also chromosome regions and bands, was proposed for the first time in 1971 at the Fourth International Congress of Human Genetics in Paris [4], later it developed into the document entitled "An International System for Human Cytogenetic Nomenclature", the last edition of which was published in 2005 [5]. Although recently evolved molecular cytogenetic techniques [6-8] and array-CGH [9] allow precise characterization of chromosomal abnormalities, analysis of cytogenetic bands is still of great importance. It is often the first step for a clinical diagnosis and in research to understand the biology of an inherited or acquired disease.

The present nomenclature of human chromosomes has been chosen in a more or less randomly manner only by morphological comparison of chromosomal G-bands at different resolution levels and without any systematic investigation about the origin of chromosomal bands. This might be the reason why mapping data from the human genome project do not always fit to the cytogenetic gene mapping data. Thus, an accurate banding nomenclature is required for precise characterization of chromosomal abnormalities.

Recent studies of metaphase chromosomes have revealed that they are remarkably elastic and can be stretched [10,11]. This extensibility of mitotic chromosomes has been used to increase the resolution of chromosome banding and to do for the first time the systematic analyses of chromosome band splitting [12,13]. It was found that new sub-bands appeared during the chromosome stretching process and that these sub-bands arose only from G-dark bands. Recently, to confirm these observations we applied another approach which analyzed behavior of multicolor banding (MCB) based pseudo-color bands in respect to G bands on human chromosome 5 using chromosome preparations of different length [14]. Here, we extended and complemented these studies and present for the first time the biologically based hierarchically organized splitting of chromosomal bands for all human chromosomes.

## Results and discussion

**Figure 1:**
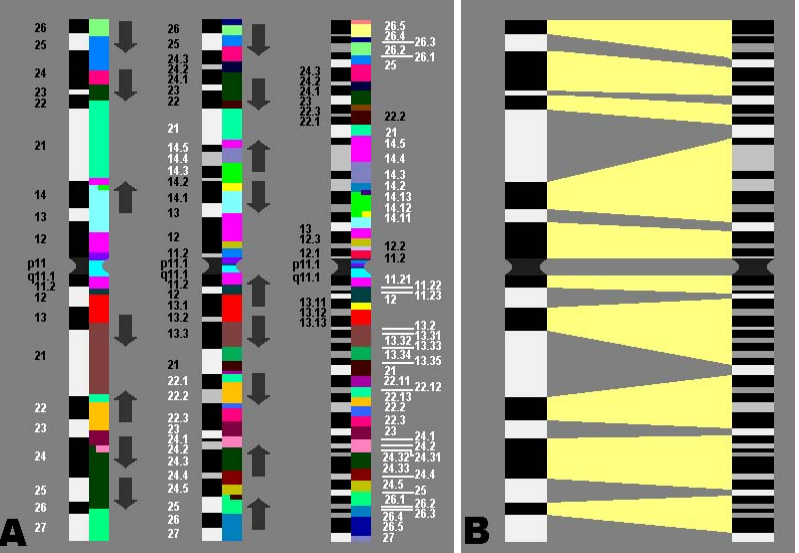
**A) Schematic summary of the aMCB results obtained on chromosome 3, each for the used three lengths of the chromosome**. For a better overview the three different aMCB banding patterns are depicted in equal sizes, each. The corresponding G banding ideograms of chromosome 3 at the 300-, 550-, or 850-band level (right side of each aMCB scheme) were used for comparison of the three different stages. The arrows indicate the chromosome swelling appearing in the different sub-bands. B) Schematic drawing depicting which regions are homologous between chromosome 3 at the 300-band stage and at the 850-band stage, as defined based on the aMCB-results. Note that only G-dark bands split into additional sub-bands

**Figure 2:**
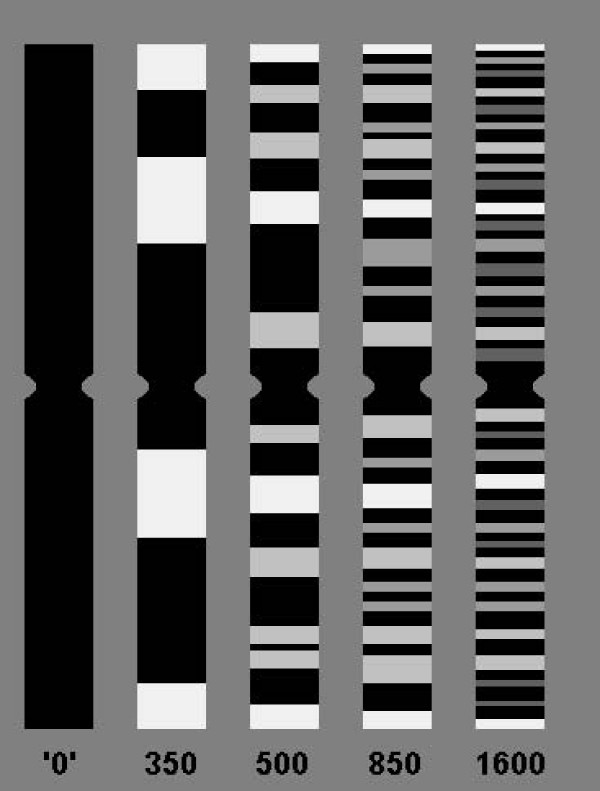
**Schematic band splitting of any chromosome; chromosomes are shown at same size in all stages; even though they would become larger the more bands show up**. At the beginning, in extremely short chromosomes, there are no bands ('0'-band stage); at 350 band-level there are a few new Giemsa light bands (white), at 500 band-level additional Giemsa light bands appear (light gray) and the initial Giemsa light bands do not further split (white) – the same happens at 850- and 1600 band-levels – new Giemsa light bands develop from Giemsa dark bands – new Giemsa light bands are depicted in gray and dark gray.

Analysis of the splitting of chromosomal bands into sub-bands during the decondensation process from 300 up to 850 bands per haploid karyotype by using array-proved MCB (aMCB) enabled to demonstrate that only G-dark bands split into dark and light chromosomal sub-bands (Figs. 1 and 2). Thus, this fact suggested before, after application of chromosome stretching was confirmed for the entire human karyotype by a second and independent approach. This makes the concept of chromosomal region specific protein swelling even more evident [15]. It has to be stressed, that the probes underlying the aMCB-approach are the only molecular cytogenetic probes which are molecular defined, i.e. they are the only within the human DNA sequence anchored FISH banding probes [16]. Thus, the probes are well defined and highly suited for such an approach as here done.

**Figure 3:**
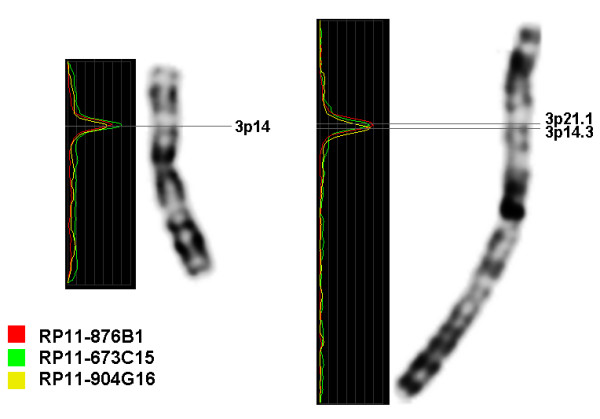
**FISH results with BAC probes**. All three BAC probes for chromosome 3 were cytogenetically located at 3p14 when hybridized to short chromosomes. On long, almost prometaphase chromosomes, we could observe that BACs RP11-876B11 and RP11-673C15 localized in the sub-band 3p21.1, that indicates that the cytogenetic band 3p21.1 actually originated from the band 3p14 and thus could be assigned as 3p14.4.

To confirm band splitting results after aMCB we selected 2 sets of BAC probes for chromosome 3 and 7 located according to UCSC Genome Browser Database in the neighboring bands 3p21.1 (RP11-876B11: 54,006,044-54,210,950 MB, RP11-673C15: 54,195,430-54,358,466 MB) and 3p14.3 (RP11-904G16: 54,423,824-54,621,639 MB), or 7q11.21 (RP11-458F8: 65,934,704-66,092,418 MB) and 7q11.22 (RP11-584N20: 66,119,511-66,292,087 MB, RP11-243C20: 66,333,985-66,515,748 MB), respectively. BACs for one chromosome were labeled with different fluorochromes and hybridized simultaneously to chromosomes of different length. After that their cytogenetic location was determined using inverted DAPI on short and long chromosomes (Fig. 3). On long chromosomes BAC probes were cytogenetically located at the sub-bands indicated in the UCSC database. At the same time, on shorter chromosomes we could localize all BAC probes for chromosome 7 at 7q11.1. This supports the band splitting data obtained by the aMCB approach, as it indicates that both sub-bands 7q11.21 and 7q11.22 arise indeed from the band 7q11.1. Thus, in a biological nomenclature they should be attributed as 7q11.2 and 7q11.3. BAC FISH results for chromosome 3 could also prove band splitting scheme for 3q14.3-3q21.1 region of chromosome 3 derived on the basis of aMCB FISH (see for the Fig. 3).

**Figure 4:**
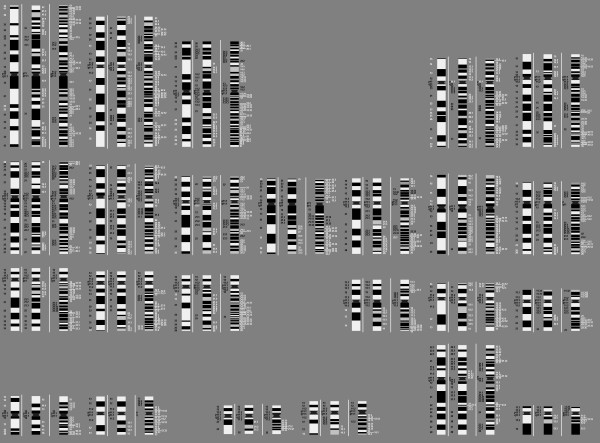
**Whole human karyotype depicted by ideograms starting at 350 band level going via 500 up to 850 bands**. Each chromosome was analyzed by multicolor banding with special regard to the splitting up of chromosomal bands into sub-bands. Black numbers are in concordance with ISCN 2005, white had to be altered to show the biological correct way of band splitting. Only G-dark bands split into additional sub-bands. This context is exemplified in a biological nomenclature proposal, which starts with the names of the bands given in ISCN 2005 at the 300 band level (see also Table 1). The white and gray colorings of the GTG-light bands correspond to the splitting of the bands as explained in the legend of Fig. 2.

Basic on our data, we suggest a biologically correct nomenclature of human chromosome band splitting. **We have to state explicitly that we do not intend to replace the ISCN nomenclature by that one, as the latter has been used now for nearly 40 years**. Changing the band names would lead to a complete mess in the cytogenetic literature. However, especially to better align new molecular with cytogenetic data the underlying biological nature of band splitting has to be known. For that we started – as far as possible with the band-nomenclature of ISCN 2005 at the 300 and/or 400 band level. However, we had to correct in some cases the ideograms – especially at the very ends of some chromosomes (e.g. 4p, 8p, and others), by including yet not shown dark bands. This is why, according to figures of all human chromosomes in different decondensation states (see [5] pp 32 to 33) there are additional GTG dark bands in the human chromosomes, not yet included in the commonly used ideograms; these yet not recognized bands are listed in table 1. The observed aMCB band-splitting could be explained only by these additional bands, which is a molecular prove for their existence besides the visual proves of GTG-banded chromosomes present in each cytogenetic laboratory (Fig. 4). Similar observations like the here reported ones were previously discussed in other DNA-based chromosome structure studies. The combinations of extreme Alu-richness and GC-richness could be aligned with GTG-/R-bands to so-called isochores [17-19].

**Table 1:** Differences of ideograms used here compared to those in ISCN 2005:

chromosome	300	550	850
1	-	-	-

2	-	-	-

3	-	-	-

4	*4p15* *4q34*	*4p15* *4q34*	-

5	**5q14–5q21**	-	*5q34.11* *5q34.12* *5q34.13* *5q34.51* *5q34.52* *5q34.53*

6	*6p26* **6q14-q16** **6q26-q27**	*6p26*	

7	*7p23* *7q35*	*7p23* *7q35.1* *7q35.2*	-

8	*8p24* *8q25*	-	-

9	*9p25* *9q33*	-	-

10	-	-	-

11	*11p16* *no 11q25*	*No splitting of 11p16.1 and 11q14.1 at this stage*	-

12	*12p14* **12q21-q23**	*12p14*	-

13	**13q31-q33**	-	-

14	**14q12-q21**	-	-

15	-	-	-

16	-	-	-

17	*17p14*	*17p14.1* *17p14.2*	*17p14.11* *17p14.12* *17p14.13* *17p14.2*

18	-	-	-

19	*19p14* *19q16*	-	-

20	-	-	-

21	-	-	-

22	*22q14*	-	-

X	**Xp11.3-p21** **Xq23-q25**	-	-

Y	-	-	-

The biologically based nomenclature from Fig. 4 is used in this table. Abbreviations:

- = no difference to ISCN 2005 ideograms

Letters in bold: Normally, the ideograms of ISCN 2005 at a 300 band level were used as starting point for biologically based band-splitting; this was not done when sub-bands are shown in ISCN 2005 as one fused band. Such bands are mentioned and depicted here in bold letters.

Letters in italics: In case new bands not mentioned at the corresponding band level but indeed visible on the chromosomes on more condensed chromosomes these are mentioned and printed here in italics.

As noted by Kowalska and coworkers (2007) [20] sub-band information provided in different human genome sequence databases are not identical. This might be partially due to the different initial sources of G-banded ideogram used and leads to discrepancies in gene mapping in different databases. ISCN ideograms are used in the NCBI Human Genome browser  and are based on morphological comparison of chromosomes at different G-band resolution level. At the same time UCSC  and Ensembl genome browsers  are based on the ideograms resulted from so called cytogenetic band prediction [21]. The latter method employed results from 9500 FISH experiments to approximate the locations of the 850 high-resolution bands, and thus, could define chromosome band lengths more precisely. Surprisingly, by applying this cytogenetic band prediction algorithm, it was shown that the lengths of the darkest G bands were consistently underestimated, while the opposite was true for the light bands. This finding might be also an indirect proof for the observation that only G-dark bands split into new G-dark and G-light sub-bands, the first ones containing hence potentially higher condensed DNA.

Finally, the present study was only in part able to enlighten the chromosomal architecture of the pericentromeric heterochromatin. It could be shown, that centromere-near subbbands arise e.g. from 1q12, 19q12, 20p11 and 20q11 but not from 9q12 or 16q11.2. Also no new information could be obtained for the heterochromatic short arms of the acrocentric chromosomes. So, neither current genome databases nor cytogenetic nomenclature attempts do consider the DNA or chromatin base of these still somehow enigmatic chromosomal structures.

The biological way of band splitting in peripheral blood lymphocytes is shown for each chromosome in Fig. 4 and for each chromosome ' [see Additional files 1, 2, 3, 4, 5, 6, 7, 8, 9, 10, 11, 12, 13, 14, 15, 16, 17, 18, 19, 20, 21, 22, 23, 24]. **Note: these ideograms are NOT intended to replace neither ISCN ideograms nor nomenclature!**

## Conclusion

In summary, we achieved now a better understanding of the chromosomal architecture (Fig. 4). The biologically correct nomenclature of sub-bands was found to be in more than 50% different from the ISCN 2005 nomenclature. This may help explaining why mapping data from the human genome project do not always fit the cytogenetic gene mapping data.

## Methods

### Metaphase preparations

Metaphase preparations were done from normal human peripheral lymphocyte cultures. To study chromosomes of different length, different cultivating protocols were applied to obtain metaphases with 300–400, 550 and 850 bands per haploid karyotype. Chromosomes at 300–400 band stages were prepared by harvesting cultures using standard cytogenetic methods [22]. Methotrexate mediated cell culture synchronization [23] was done to prepare chromosomes at 550-band stage. In order to obtain chromosome preparations at 850-band level we used Synchroset (Euroclone), in combination with Chromosome Kit P (Euroclone) and Buffered Hypotonic Solution (ProCell Reagents).

### Multicolor banding (aMCB)

Recently BAC-array mapped aMCB probe sets [16] for all human chromosomes were applied according to standard protocols [24,25]. For evaluation of the fluorescence in situ hybridization (FISH) results, ISIS software (MetaSystems, Altlussheim, Germany) was used acc. [14].

### Analysis of aMCB

Using ISIS software (MetaSystems, Altlussheim, Germany) chromosome region-specific fluorescence profiles can be converted into computer-based pseudo-colors. One pseudo-color band corresponds to a specific fluorochrome combination and (in parts) to a specific fluorochrome intensity, which can be variably assigned to any resolution level. As stated in [14], pseudo-color schemes with different number of pseudo-colors were created at the G-stage of 850 bands, and then applied to chromosomes of different length. When limited number of pseudo-colors is used, aMCB pattern remains stable irrespective of the chromosome condensation [14]. But if higher numbers of pseudo-colors are assigned, then disappearance of some pseudo-colors was observed on middle (550 bands stage) and short (300–400 bands stage) chromosomes. 10 copies of each chromosome at each band-stage level were evaluated; the evaluation process followed the rules suggested previously by [14].

### BAC clones

BAC clones, RP11-876B11, RP11-673C15, RP11-904G16, RP11-458F8, RP11-584N20, RP11-243C20 were purchased from Sanger Centre, UK  DNA preparations from cultured bacteria (containing required construct) were performed using the QIAprep Spin Miniprep Kit (Qiagen) according to the manufacturer's protocol. DNA was amplified using DOP-PCR with three low-temperature cycles, and then labelled with biotin-16-dUTP, SpectrumGreen-dUTP or SpectrumOrange-dUTP by label-PCR [25,26]. Biotin-16-dUTP labeled probes were detected with Streptavidin-Cy5. Unincorporated nucleotides were removed by ethanol precipitation. BACs for the same chromosome were hybridized in parallel to chromosome preparations of different length.

## Competing interests

The authors declare that they have no competing interests.

## Authors' contributions

NK and HN performed the necessary aMCB studies. NK and AW did the BAC-FISH selection and studies. UC, AW, TL have been involved in drafting the manuscript and revising it critically for important intellectual content.

## Supplementary Material

Additional file 1 **Band-splitting of chromosome 1**. Biological band-splitting of chromosome 1 as observed in peripheral lymphocytes. Click here for file

Additional file 2 **Band-splitting of chromosome 2**. Biological band-splitting of chromosome 2 as observed in peripheral lymphocytes. Click here for file

Additional file 3 **Band-splitting of chromosome 3**. Biological band-splitting of chromosome 3 as observed in peripheral lymphocytes. Click here for file

Additional file 4 **Band-splitting of chromosome 4.** Biological band-splitting of chromosome 4 as observed in peripheral lymphocytes. Click here for file

Additional file 5 **Band-splitting of chromosome 5**. Biological band-splitting of chromosome 5 as observed in peripheral lymphocytes. Click here for file

Additional file 6 **Band-splitting of chromosome 6**. Biological band-splitting of chromosome 6 as observed in peripheral lymphocytes. Click here for file

Additional file 7 **Band-splitting of chromosome 7**. Biological band-splitting of chromosome 7 as observed in peripheral lymphocytes. Click here for file

Additional file 8 **Band-splitting of chromosome 8. **Biological band-splitting of chromosome 8 as observed in peripheral lymphocytes. Click here for file

Additional file 9 **Band-splitting of chromosome 9**. Biological band-splitting of chromosome 9 as observed in peripheral lymphocytes. Click here for file

Additional file 10 **Band-splitting of chromosome 10**. Biological band-splitting of chromosome 10 as observed in peripheral lymphocytes. Click here for file

Additional file 11 **Band-splitting of chromosome 11**. Biological band-splitting of chromosome 11 as observed in peripheral lymphocytes. Click here for file

Additional file 12 **Band-splitting of chromosome 12**. Biological band-splitting of chromosome 12 as observed in peripheral lymphocytes. Click here for file

Additional file 13 **Band-splitting of chromosome 13**. Biological band-splitting of chromosome 13 as observed in peripheral lymphocytes. Click here for file

Additional file 14 **Band-splitting of chromosome 14**. Biological band-splitting of chromosome 14 as observed in peripheral lymphocytes. Click here for file

Additional file 15 **Band-splitting of chromosome 15**. Biological band-splitting of chromosome 15 as observed in peripheral lymphocytes. Click here for file

Additional file 16 **Band-splitting of chromosome 16**. Biological band-splitting of chromosome 16 as observed in peripheral lymphocytes. Click here for file

Additional file 17 **Band-splitting of chromosome 17**. Biological band-splitting of chromosome 17 as observed in peripheral lymphocytes. Click here for file

Additional file 18 **Band-splitting of chromosome 18.** Biological band-splitting of chromosome 18 as observed in peripheral lymphocytes. Click here for file

Additional file 19 **Band-splitting of chromosome 19**. Biological band-splitting of chromosome 19 as observed in peripheral lymphocytes. Click here for file

Additional file 20 **Band-splitting of chromosome 20**. Biological band-splitting of chromosome 20 as observed in peripheral lymphocytes. Click here for file

Additional file 21 **Band-splitting of chromosome 21**. Biological band-splitting of chromosome 21 as observed in peripheral lymphocytes. Click here for file

Additional file 22 **Band-splitting of chromosome 22**. Biological band-splitting of chromosome 22 as observed in peripheral lymphocytes. Click here for file

Additional file 23 **Band-splitting of the X-chromosome**. Biological band-splitting of the X-chromosome as observed in peripheral lymphocytes. Click here for file

Additional file 24 **Band-splitting of the Y-chromosome.** Biological band-splitting of the Y-chromosome as observed in peripheral lymphocytes. Click here for file

## References

[B1] Caspersson T, Zech L, Johansson C (1970). Differential binding of alkylating fluorochromes in human chromosomes. Exp Cell Res.

[B2] Seabright M (1971). A rapid banding technique for human chromosomes. Lancet.

[B3] Drets ME, Shaw MW (1971). Specific banding patterns of human chromosomes. Proc Nat Acad Sci USA.

[B4] The National Foundation (1972) Paris Conference 1971. Standardization in human cytogenetics.

[B5] Shaffer LG, Tommerup N, ISCN 2005 (2005). An international system for cytogenetic nomenclature. S Karger, Basel, Switzerland.

[B6] Speicher MR, Gwyn Ballard S, Ward DC Karyotyping human chromosomes by combinatorial multi-fluor FISH. Nature Genet.

[B7] Schröck E, du Manoir S, Veldman T, Schoell B, Wienberg J, Ferguson-Smith MA, Ning Y, Ledbetter DH, Bar-Am I, Soenksen D, Garini Y, Ried T (1996). Multicolor spectral karyotyping of human chromosomes. Science.

[B8] Liehr T, Starke H, Heller A, Kosyakova N, Mrasek K, Gross M, Karst C, Steinhaeuser U, Hunstig F, Fickelscher I, Kuechler A, Trifonov V, Romanenko SA, Weise A (2006). Multicolor fluorescence in situ hybridization (FISH) applied to FISH-banding. Cytogenet Genome Res.

[B9] Pinkel D, Segraves R, Sudar D, Clark S, Poole I, Kowbel D, Collins C, Kuo WL, Chen C, Zhai Y, Dairkee SH, Ljung BM, Gray JW, Albertson DG (1998). High resolution analysis of DNA copy number variation using comparative genomic hybridization. Nature Genet.

[B10] Claussen U, Mazur A, Rubtsov N (1994). Chromosomes are highly elastic and can be stretched. Cytogenet Cell Genet.

[B11] Marko JF (2008). Micromechanical studies of mitotic chromosomes. Chromosome Res.

[B12] Hliscs R, Mühlig P, Claussen U (1997). The nature of G-bands analyzed by chromosome stretching. Cytogenet Cell Genet.

[B13] Kuechler A, Mueller CR, Liehr T, Claussen U (2001). Detection of microdeletions in the short arm of the X chromosome by chromosome stretching. Cytogenet Cell Genet.

[B14] Lehrer H, Weise A, Michel S, Starke H, Mrasek K, Heller A, Kuechler A, Claussen U, Liehr T (2004). The hierarchically organized splitting of chromosome bands into sub-bands analyzed by multicolor banding (MCB). Cytogenet Genome Res.

[B15] Claussen U, Michel S, Mühlig P, Westermann M, Grummt U-W, Kromeyer-Hauschild K, Liehr T (2002). Demystifying chromosome preparation and the implications fort he concept of chromosome condensation during mitosis. Cytogenet Cell Genet.

[B16] Weise A, Mrasek K, Fickelscher I, Claussen U, Cheung SW, Cai WW, Liehr T, Kosyakova N (2008). Molecular definition of high-resolution multicolor banding probes: first within the human DNA sequence anchored FISH banding probe set. J Histochem Cytochem.

[B17] Holmquist GP (1992). Chromosome bands, their chromatin flavors, and their functional features. Am J Hum Genet.

[B18] Holmquist GP, Ashley T (2006). Chromosome organization and chromatin modification: influence on genome function and evolution. Cytogenet Genome Res.

[B19] Korenberg JR, Rykowski MC (1988). Human genome organization: Alu, lines, and the molecular structure of metaphase chromosome bands. Cell.

[B20] Kowalska A, Bozsaky E, Ramsauer T, Rieder D, Bindea G, Lörch T, Trajanoski Z, Ambros PF (2007). A new platform linking chromosomal and sequence information. Chromosome Res.

[B21] Furey TS, Haussler D (2003). Integration of the cytogenetic map with the draft human genome sequence. Hum Mol Genet.

[B22] Verma RS, Babu A (1995). Human chromosomes: Principles and techniques.

[B23] Yunis JJ (1976). High resolution of human chromosomes. Science.

[B24] Chudoba I, Plesch A, Lörch T, Lemke J, Claussen U, Senger G (1999). High resolution multicolor-banding: a new technique for refined FISH analysis of human chromosomes. Cytogenet Cell Genet.

[B25] Liehr T, Heller A, Starke H, Rubtsov N, Trifonov V, Mrasek K, Weise A, Kuechler A, Claussen U (2002). Microdissection based high resolution multicolor banding for all 24 human chromosomes. Int J Mol Med.

[B26] Liehr T, Weise A, Heller A, Starke H, Mrasek K, Kuechler A, Weier HU, Claussen U (2002). Multicolor chromosome banding (MCB) with YAC/BAC-based probes and region-specific microdissection DNA libraries. Cytogenet Genome Res.

