# Supplementary figures and images for: The hierarchically organized splitting of chromosomal bands for all human chromosomes
Source: Mol Cytogenet. 2009 Jan 26;2:4. doi: 10.1186/1755-8166-2-4 (PMC2636822; doi:10.1186/1755-8166-2-4)

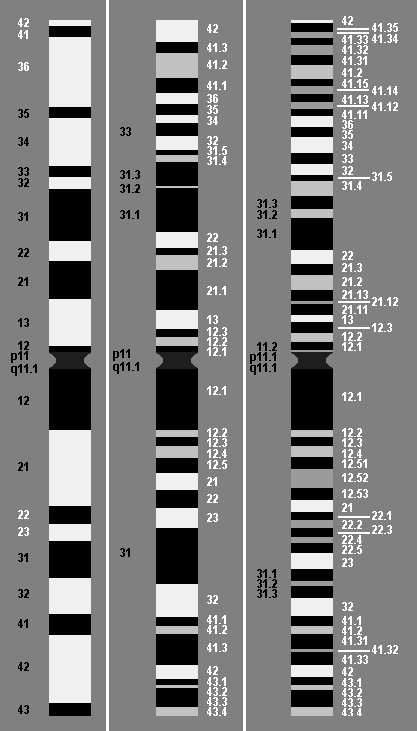

Supplement: Additional file 1 — Band-splitting of chromosome 1. Biological band-splitting of chromosome 1 as observed in peripheral lymphocytes. [file 1755-8166-2-4-S1.tiff]

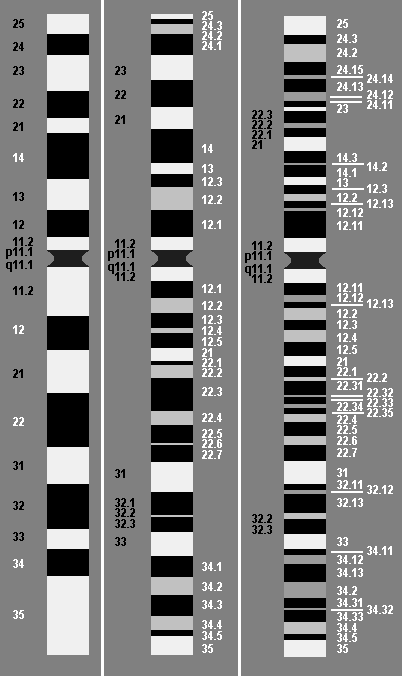

Supplement: Additional file 2 — Band-splitting of chromosome 2. Biological band-splitting of chromosome 2 as observed in peripheral lymphocytes. [file 1755-8166-2-4-S2.tiff]

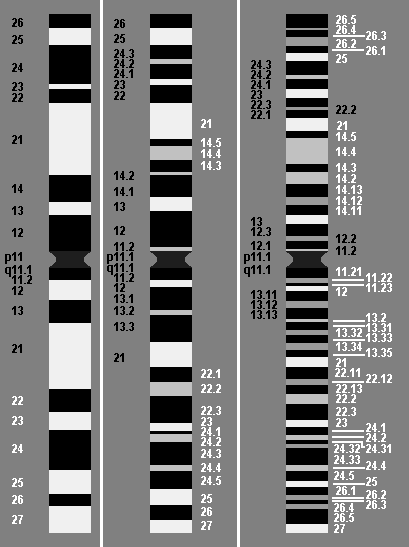

Supplement: Additional file 3 — Band-splitting of chromosome 3. Biological band-splitting of chromosome 3 as observed in peripheral lymphocytes. [file 1755-8166-2-4-S3.tiff]

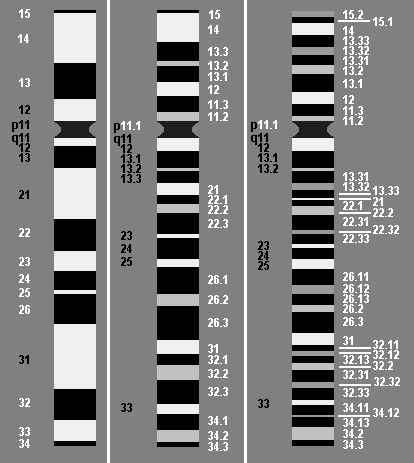

Supplement: Additional file 4 — Band-splitting of chromosome 4. Biological band-splitting of chromosome 4 as observed in peripheral lymphocytes. [file 1755-8166-2-4-S4.tiff]

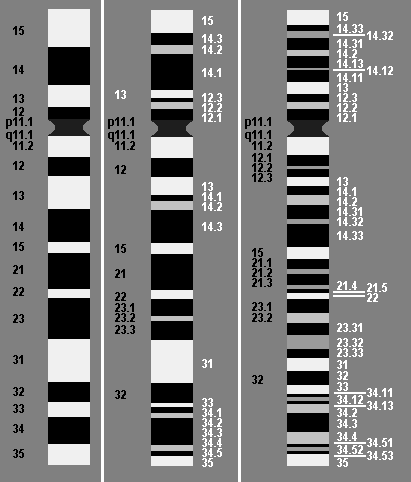

Supplement: Additional file 5 — Band-splitting of chromosome 5. Biological band-splitting of chromosome 5 as observed in peripheral lymphocytes. [file 1755-8166-2-4-S5.tiff]

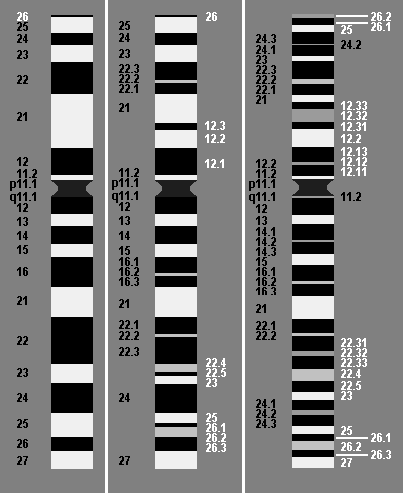

Supplement: Additional file 6 — Band-splitting of chromosome 6. Biological band-splitting of chromosome 6 as observed in peripheral lymphocytes. [file 1755-8166-2-4-S6.tiff]

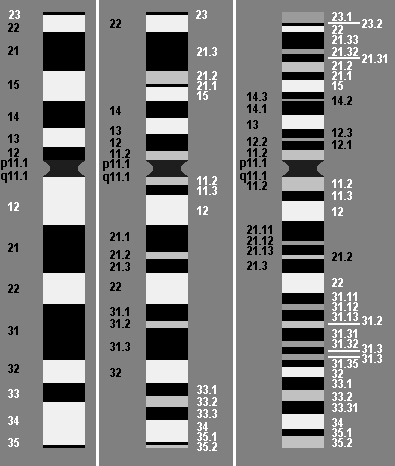

Supplement: Additional file 7 — Band-splitting of chromosome 7. Biological band-splitting of chromosome 7 as observed in peripheral lymphocytes. [file 1755-8166-2-4-S7.tiff]

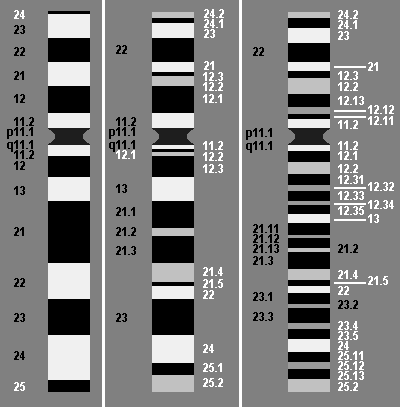

Supplement: Additional file 8 — Band-splitting of chromosome 8. Biological band-splitting of chromosome 8 as observed in peripheral lymphocytes. [file 1755-8166-2-4-S8.tiff]

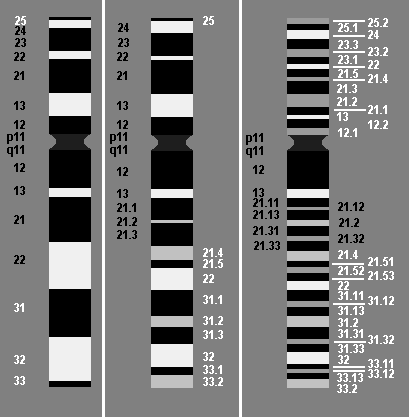

Supplement: Additional file 9 — Band-splitting of chromosome 9. Biological band-splitting of chromosome 9 as observed in peripheral lymphocytes. [file 1755-8166-2-4-S9.tiff]

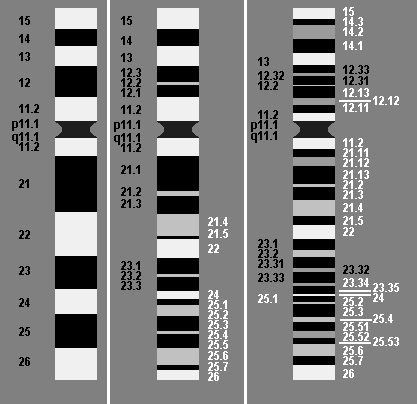

Supplement: Additional file 10 — Band-splitting of chromosome 10. Biological band-splitting of chromosome 10 as observed in peripheral lymphocytes. [file 1755-8166-2-4-S10.tiff]

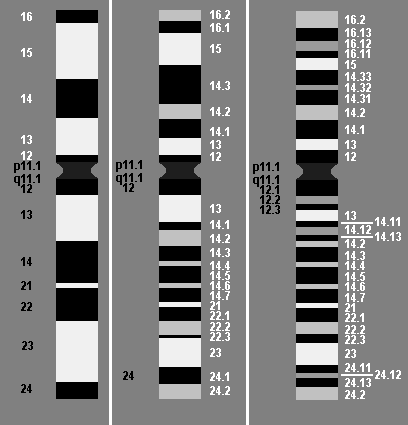

Supplement: Additional file 11 — Band-splitting of chromosome 11. Biological band-splitting of chromosome 11 as observed in peripheral lymphocytes. [file 1755-8166-2-4-S11.tiff]

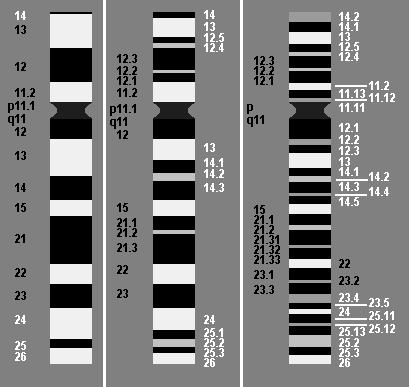

Supplement: Additional file 12 — Band-splitting of chromosome 12. Biological band-splitting of chromosome 12 as observed in peripheral lymphocytes. [file 1755-8166-2-4-S12.tiff]

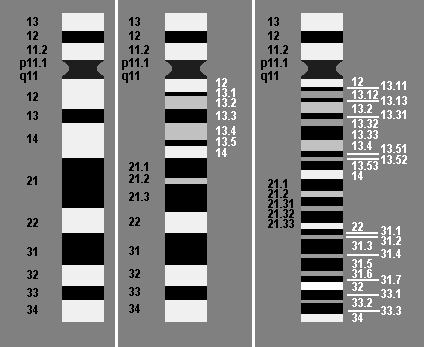

Supplement: Additional file 13 — Band-splitting of chromosome 13. Biological band-splitting of chromosome 13 as observed in peripheral lymphocytes. [file 1755-8166-2-4-S13.tiff]

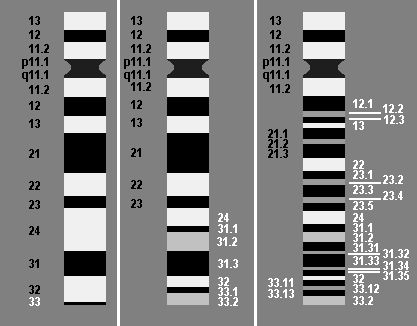

Supplement: Additional file 14 — Band-splitting of chromosome 14. Biological band-splitting of chromosome 14 as observed in peripheral lymphocytes. [file 1755-8166-2-4-S14.tiff]

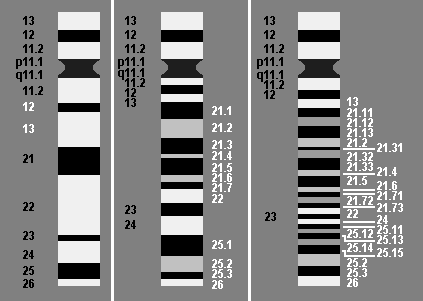

Supplement: Additional file 15 — Band-splitting of chromosome 15. Biological band-splitting of chromosome 15 as observed in peripheral lymphocytes. [file 1755-8166-2-4-S15.tiff]

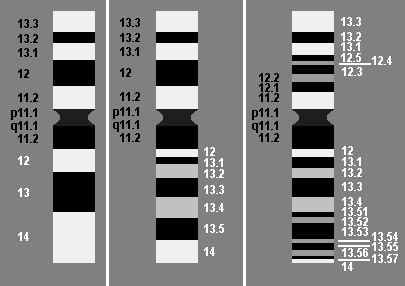

Supplement: Additional file 16 — Band-splitting of chromosome 16. Biological band-splitting of chromosome 16 as observed in peripheral lymphocytes. [file 1755-8166-2-4-S16.tiff]

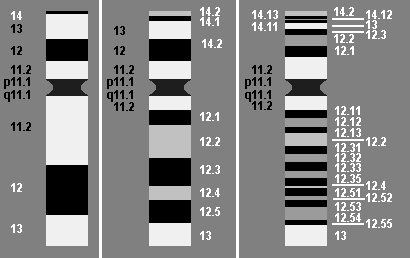

Supplement: Additional file 17 — Band-splitting of chromosome 17. Biological band-splitting of chromosome 17 as observed in peripheral lymphocytes. [file 1755-8166-2-4-S17.tiff]

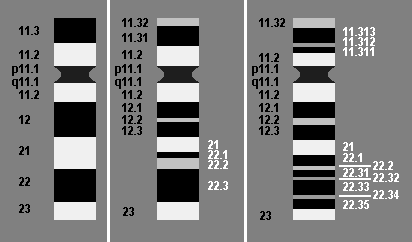

Supplement: Additional file 18 — Band-splitting of chromosome 18. Biological band-splitting of chromosome 18 as observed in peripheral lymphocytes. [file 1755-8166-2-4-S18.tiff]

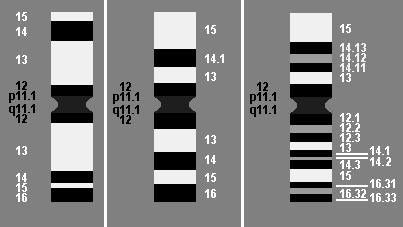

Supplement: Additional file 19 — Band-splitting of chromosome 19. Biological band-splitting of chromosome 19 as observed in peripheral lymphocytes. [file 1755-8166-2-4-S19.tiff]

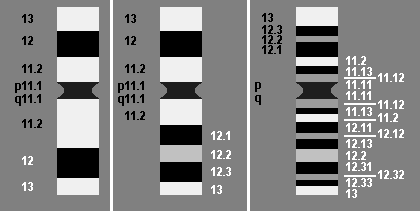

Supplement: Additional file 20 — Band-splitting of chromosome 20. Biological band-splitting of chromosome 20 as observed in peripheral lymphocytes. [file 1755-8166-2-4-S20.tiff]

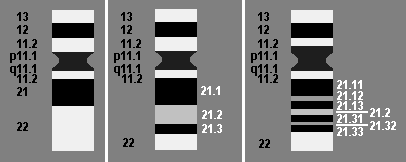

Supplement: Additional file 21 — Band-splitting of chromosome 21. Biological band-splitting of chromosome 21 as observed in peripheral lymphocytes. [file 1755-8166-2-4-S21.tiff]

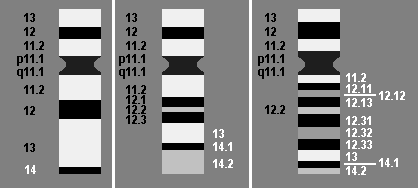

Supplement: Additional file 22 — Band-splitting of chromosome 22. Biological band-splitting of chromosome 22 as observed in peripheral lymphocytes. [file 1755-8166-2-4-S22.tiff]

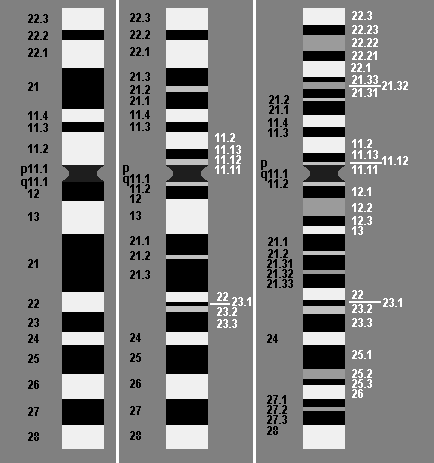

Supplement: Additional file 23 — Band-splitting of the X-chromosome. Biological band-splitting of the X-chromosome as observed in peripheral lymphocytes. [file 1755-8166-2-4-S23.tiff]

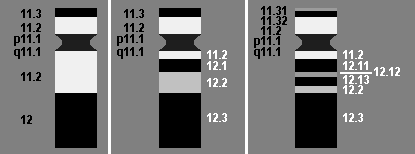

Supplement: Additional file 24 — Band-splitting of the Y-chromosome. Biological band-splitting of the Y-chromosome as observed in peripheral lymphocytes. [file 1755-8166-2-4-S24.tiff]
